# Physicians Visiting List

**Published:** 1853-01

**Authors:** 


### PHYSICIAN'S VISITING LIST.

The publishers last mentioned have done the profession a service by issuing this little work, which is at once a visiting list, a diary, and a book of engagements. We recommend it to all our brethren, and believe that after using it they will not be willing to be again without it.

